# Gas-Induced Structural Damages in Forward-Bias Bipolar Membrane CO_2_ Electrolysis Studied by Fast X-ray Tomography

**DOI:** 10.1021/acsaem.3c02882

**Published:** 2024-04-19

**Authors:** Robert Fischer, Matthieu A. Dessiex, Federica Marone, Felix N. Büchi

**Affiliations:** † Electrochemistry Laboratory, Paul Scherrer Institut, 5232 Villigen PSI, Switzerland; ‡ Laboratory of Renewable Energy Science and Engineering, Ecole Polytechnique Fédérale de Lausanne (EPFL), 1015 Lausanne, Switzerland; § Swiss Light Source, Paul Scherrer Institut, 5232 Villigen PSI, Switzerland

**Keywords:** CO_2_ electrolysis, bipolar membranes, X-ray tomographic microscopy, electrochemical CO_2_ reduction, degradation, synchrotron

## Abstract

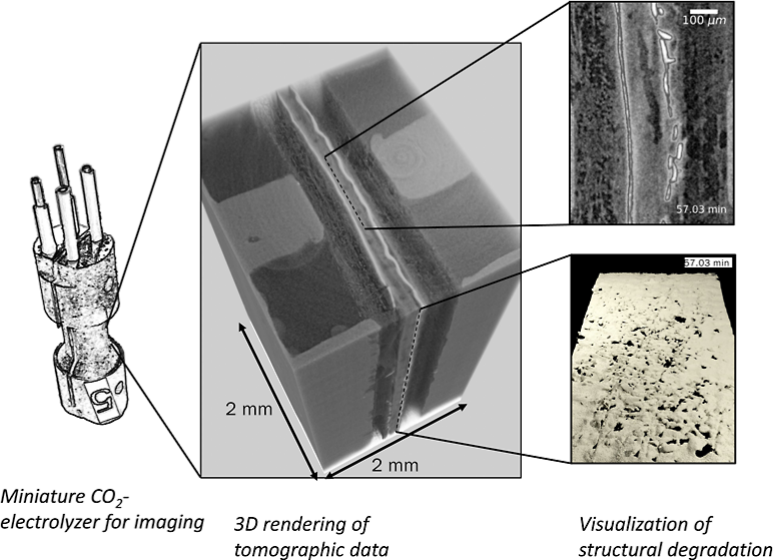

Forward-bias bipolar membrane (BPM) CO_2_ coelectrolysis (CO2ELY) aims at overcoming the issues of salt precipitation and CO_2_ crossover in anion exchange membrane CO2ELY. Increasing the stability of BPM-CO2ELY is crucial for widespread application of the technique. In this study, we employ time-resolved X-ray tomographic microscopy to elucidate the structural dynamics that occur within the electrochemical cell during operation under various conditions. Using advanced image processing methods, including custom 4D machine learning segmentation, we can visualize and quantify damages in the membrane and anode catalyst layer (CL). We compare our results to a CO_2_ transport model and hypothesize gaseous CO_2_ as the cause of the observed damages. At any operation condition, CO_2_ is formed at the junction in the center of the BPM by recombination of carbonate ions. CO_2_ migrates to the anode by diffusion and goes into the gas phase at the interface of the membrane and anode CL. After sufficient CO_2_ accumulation and pressure buildup after only tens of minutes, small irreversible holes break into the CL distributed over the entire active area. Additionally, at higher current densities, the CO_2_ accumulation leads to membrane delamination at the BPM junction. Despite the clear degradation processes, we do not observe an obvious direct effect on the electrochemical performance. The poor stability of BPM-CO2ELY remains an open question.

## Introduction

Electrochemical reduction of CO_2_ and water via coelectrolysis (CO2ELY) is a promising technique to utilize electric energy for nonfossil production of chemical feedstock and utilization of captured CO_2_.^1^ In this study, we consider the reduction of CO_2_ to CO on silver in an alkaline medium at the cathode side of the cell with the hydrogen evolution as a competing reaction
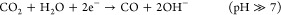
1a
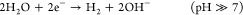
1b

Regardless of which reaction dominates, the same amount of hydroxide is produced per electron. At the anode, oxygen is evolved as in alkaline or acidic water electrolysis
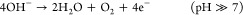
2a
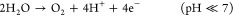
2b

Practical application of CO2ELY at a large scale currently lacks the required current density and stability to be economically feasible.^2^ Gas-fed CO_2_-electrolyzers in a zero-gap architecture with an anion exchange membrane (AEM) are considered to be the most promising architecture.^3^ However, AEM CO2ELY has two major issues: CO_2_ crossover and salt precipitation. The CO_2_ crossover in alkaline media is driven by the chemical reaction of CO_2_ with hydroxide (OH^–^) to form (bi)carbonate ions^4^
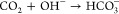
3a
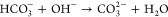
3b

The (bi)carbonate formation is not an electrochemical reaction, does not directly depend on current density or potential, and occurs regardless of the Faradaic efficiency (equations 1 and 3). The carbonate ions partly replace the hydroxides as the charge carriers in the AEM are conducted to the anode^4^ and electrochemically react to oxygen and CO_2_
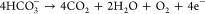
4a
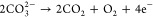
4b

The CO_2_-crossover presents an energy penalty caused by the downstream separation of CO_2_^5,6^ to be reused in the process. Salt precipitation at the cathode occurs in the form of carbonate salts, e.g., K_2_CO_3_. (Bi)carbonate anions are formed at the cathode (reaction equation). While the AEM is in principle is permselective, cations like K^+^ can still migrate to the cathode and form carbonate salts.^6^ Alkali metal cations from the anode reach the cathode by electro-osmotic diffusion due to gradients in electric potential and concentration. Empirical data suggest the need of an alkaline electrolyte for good performance and stability, e.g., KOH or KHCO_3_, at the anode.^3^ Recent studies could furthermore show that sufficient direct supply of alkali cations at the cathode improves performance and stability.^7−9^ Effectively, cations not only have an apparent positive effect on the CO_2_ reduction reaction but also lead to salt precipitation and fast performance degradation, calling for a delicate balance^9,10^ or regularly regenerating the cathode.^7^ The effect of cations on the CO_2_ reduction kinetics is currently a discussed topic in the field of CO2ELY, including the question whether modifying ionomers in the catalyst composition might potentially achieve the same functionality.^9^ The salt precipitates can draw electrolytes from the anode by hygroscopy to the cathode, causing flooding and further enhancing salt precipitation.^11^ Ultimately, the precipitated salt blocks the catalyst and gas diffusion layers (GDLs) and prevents CO_2_ reduction.^11^ Carbonate salt formation can be severe in only few hours of operation.^12^

Employing a bipolar membrane (BPM) in the forward bias tackles both the issues. A BPM is composed of an anion exchange layer (AEL) and a cation exchange layer (CEL). In forward bias, the AEL faces the cathode and therefore establishes the same conditions for CO_2_ reduction as in AEM-CO2ELY. The need for an alkaline electrolyte at the anode that contains cations is eliminated, allowing the use of pure water for the oxygen evolution reaction.^4,13,14^ Cations other than protons are eliminated, and the salt formation at the cathode is prevented. Only a BPM allows us to set the cathode to alkaline and the anode to acidic conditions in the zero-gap cell architecture. Therefore, having an alkaline environment at the cathode for a high Faradaic efficiency for the CO production (reaction equation), low crossover, and elimination of the salt precipitation, a BPM setup in forward bias is a promising solution. BPM-CO2ELY reduces the CO_2_ crossover seen in AEM-CO2ELY because the carbonate ions in the BPM cannot cross the AEL–CEL junction in the BPM.^4^ Carbonate ions react at the junction with the protons in the CEL forming CO_2_ and water, while hydroxide ions form only water
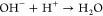
5a
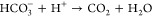
5b
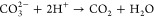
5c

While BPM-CO2ELY is in principle promising, performance degrades fast in less than 1 h of operation.^14^ Degradation processes in BPM-CO2ELY have rarely been investigated and are poorly understood. Cathode flooding^14,15^ similar to AEM-CO2ELY and polymer electrolyte fuel cells (PEMFCs),^15^ catalyst poisoning,^14^ or membrane blistering^13,16,17^ have been reported as potential candidates.

Recent *operando* studies^18^ have attempted to illuminate degradation processes in CO2ELY by employing imaging in a wider sense including neutron radiography,^11,19^ wide-angle X-ray scattering,^9,10^ or photography of an transparent cell.^12^ However, these studies are mainly concerned with the salt and flooding issues in AEM-COELY while also being limited to either two-dimensional (2D) projections or surface observation. *Operando* three-dimensional (3D) imaging methods have successfully been employed to study the performance-limiting factors in the form of two-phase flow phenomena in PEMFCs^20,21^ or water electrolysis.^22^ Time-resolved X-ray tomographic microscopy (XTM) is an established and powerful technique to study complex and 3D structural and interfacial dynamics in materials at a high spatial and temporal resolution.^23−25^ XTM can be applied *operando*, i.e., on fully operating CO2ELY cells, but typically only a synchrotron source delivers sufficient X-ray flux for high temporal resolution.

In this study, we employ time-resolved XTM to identify and characterize degradation processes in forward-bias BPM CO_2_ coelectrolysis (BPM-CO2ELY).

## Experiment

### Materials

The membrane electrode assembly (MEA) was adapted from ref (14) with the same materials if not stated differently. The cathode catalyst ink is composed of 92 wt % silver nanoparticles (APS 20–40 nm, Alfa Aesar, Schiltigheim, France) mixed in an anion conducting ionomer (FAA-3, Fumatech, Bietigheim-Bissingen, Germany). The ionomer was initially exchanged from the bromine to the carbonate form by soaking it in a 0.5 M KHCO_3_ solution for 12 h and then dissolved at 2.5 wt % in ethanol. In preparation for spray coating, the nanoparticle and ionomer were further dispersed in 50–50 vol % ultrapure water and isopropanol. The cathode catalyst ink was sprayed with an Ag loading of 2.5 mg/cm^2^ onto the microporous layer (MPL) of a carbon paper GDL (H23C8, Freudenberg, Weinheim, Germany) to form the cathode electrode. The anode catalyst ink is composed of 90 wt % IrO_2_–TiO_2_ nanoparticles (Elyst Ir75 0480, Umicore, Brussels, Belgium) in a proton conducting ionomer (5 wt % Nafion dispersion in aliphatic alcohol/water, EW = 1100 g/mol, DuPont, purchased from Sigma-Aldrich). For spraying, the IrO_2_ nanoparticles and ionomer were further dispersed in 80–20 vol % ultrapure water and isopropanol. The anode catalyst ink was sprayed on the cation exchange side of a commercial BPM (Fumasep FBM, Fumatech). The membrane is about 130 μm thick and features a woven poly(ether ether ketone) (PEEK) reinforcement mesh. An ExactaCoat (Sono-Tek, Milton, USA) machine was used to spray both inks using a custom protocol. The porous transport layer (PTL) at the anode, typically made from titanium,^4,14^ was replaced by untreated carbon paper (Sigracet 29 AA, SGL Carbon, Wiesbaden, Germany). Carbon has a higher X-ray transmittance than that of titanium and therefore helps to minimize image artifacts. Carbon paper is not the optimal choice as a PTL^26^ but retains the required water permeability and electric conductivity as a porous electrode for the duration of the experiment.

### Operando Setup

**Figure 1:**
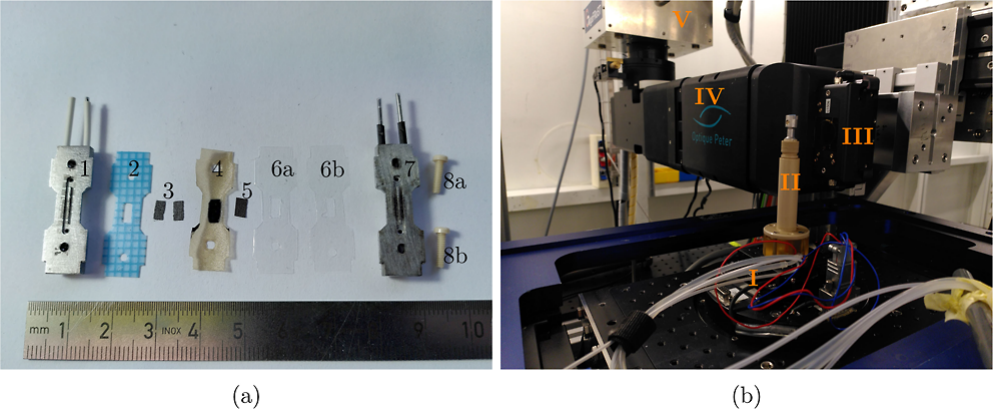
(a) Components of the miniature CO2ELY electrolyzer cell for X-ray imaging: 1-anode flow field, 2-Ice Cube gasket, 3-anode PTL (2x SGL 29 AA), 4-bipolar membrane with anode catalyst, 5-cathode GDL with CL, 6ab-FEP gaskets, 7-cathode flow field, 8ab-PEEK screws. (b) Electrolyzer mounted on the rotating sample stage at the TOMCAT beamline: I-tubing and wiring, II-sample holder, III-scintillator, IV-lens system, V-camera.

A custom miniature electrolyzer cell was devised based on a previous design for water electrolysis.^22^ The components are shown in Figure 1a. The two polar plates that contain the flow field with two channels were produced from an aluminum alloy by additive manufacturing (Feramic AG, Stallikon, Switzerland) to flange the MEA and gaskets with two nonconducting PEEK screws (Nabeya Bitech Kaisha, Seki City, Japan). Flow fields for a laboratorycoelectrolyzer are typically made from gold-coated stainless steel. However, both materials would highly attenuate X-ray and negatively impact image quality. Aluminum is a good compromise of X-ray transparency, electric conductivity, and resistance to the harsh conditions especially at the anode. The two straight channels are each 0.8 mm wide and 0.5 mm deep separated by a 0.2 mm wide rib. The width and depth of the channels are comparable to lab fixtures.^14^ Channels in lab fixtures are longer and more numerous.^14^ The gasket at the anode was 350 μm Ice Cube (35 FC-PO 100, Freudenberg), while two 75 μm thick fluorinated ethylene propylene (FEP) sheets (DuPont, Wilmington, USA) were employed at the cathode to enable sealing and reasonable compression of the GDLs to ensure electric conductivity. Lab fixtures typically employ fiber-reinforced mats of a fluorinated polymer as gaskets with the same functionality but more robustness to repeated use and higher stiffness, requiring more elaborated clamping to achieve cell tightness.^14^ The membrane was sandwiched between the gaskets that housed the GDLs with a determined active area of 2 mm by 5 mm, compared to 20 mm by 20 mm of a lab fixture.^14^ Each polar plate contains two small holes for pins to connect wires to the potentiostat (SP-300, Biologic, Seyssinet-Pariset, France). The potentiostat was connected to a personal computer containing EC-Lab software (Biologic) to control and record the electrochemical operation. Stainless steel capillaries were pressed into openings in the polar plates (Figure S1) to allow the attachment of soft tubes for gas and water streams. Since all connections are located on the same side, the electrolyzer can be mounted compactly on a rod-like sample holder onto the rotating stage for X-ray acquisition (Figure 1b). Water was heated to 65 °C and pumped in a closed cycle through the cell at the anode. The effective temperature of the cell was measured ex situ to 45 °C using a thermocouple inserted into the cathode gas inlet of the polar plate. The CO_2_ stream at the cathode was controlled by a mass flow controller (EL-FLOW Select, Bronkhorst, AK Ruurlo, the Netherlands) and was humidified in a fritted bubbler with deionized (DI) water at room temperature (23 °C) before being fed to the cell. The cathode outlet was diluted with nitrogen and led to the beamline exhaust to avoid any accumulation of H_2_ or CO.

### *Operando* Imaging and Electrochemistry Protocol

Before the cell operation was started, three pre-*operando* scans were taken. First, a full tomographic scan was taken for the mounted cell. The second scan was taken while streaming only humidified CO_2_ to the cathode. The third scan was acquired with additionally circulating water at the anode before applying any voltage. If not noted otherwise, the CO_2_ gas flux was set to 7.5 mL/min. Normalizing the gas flow to the cross-section of the two channels and active area results in 937.5 mL/cm^2^ min and 75 mL/cm^2^ min, respectively, with an estimated mean flow velocity of 0.16 m/s. Scaling the flow rate to the number of channels gives a comparable gas velocity to that of lab fixtures.^14^ Time-resolved XTM was started together with applying the first voltage to the sample. The same initial conditioning protocol was applied to all samples at constant voltage with the first 5 min at 2 V followed by 5 min at 2.5 V. Immediately after conditioning, three types of individual sample operations were performed: constant current, constant voltage, or current ramp-up. Every 5 min, electrochemical impedance spectroscopy was performed. The frequency ranged from 7 MHz to 1 Hz in 6 steps per decade in logarithmic spacing. The amplitude was 10 mV for operation at constant voltage and 100 μA for operation at constant current. The acquisition of each spectrum took about 1 min. At 100 mA/cm^2^, the cell would ideally produce 0.1 μmol/s of product gases, which is potentially detectable by available techniques. Faradaic efficiency is not the focus of this study, and we assume that the general performance is in qualitative agreement with the reported performance of a closely comparable laboratory sized CO_2_ coelectrolyzer^14^ to reduce the technical challenges of experiments at the beamline.

### Time-Resolved XTM

XTM experiments were performed at the TOMCAT-beamline of the Swiss Light Source (SLS), Paul Scherrer Institut, Switzerland. A bending magnet installed in the SLS ring provided a polychromatic X-ray beam.^27^ The beam was then filtered to the so-called 5% white beam condition with 20 mm of Sigradur and 75 μm molybden to reduce low energies and limit the overall dose to the sample. The X-rays transmitted by the sample were converted to visible light with a scintillator, whose image was recorded using the GigaFRoST^28^ camera mounted on a macroscope.^29^ To record a tomographic image, 1000 projections were recorded, while the sample was rotating by 180°. The acquisition of one projection took 1 ms resulting in 1 s for one full tomographic scan. To avoid winding up the connected tubes and cables, the sample was rotated back to its initial position before acquiring the next scan. The fast shutter was closed between two scans to limit the dose. Typically, one scan was recorded every minute for the first 45 min of CO2ELY operation followed by variable sequences selected based on the electrochemical performance. Before each sequence, 10 dark-field (no beam) and 200 flat-field (with beam, no sample) images were recorded to correct for background heterogeneities. The projections were Paganin filtered^30^ and reconstructed.^31^ Detailed parameters for the image acquisition and reconstruction are given in Table 1. One test sample was operated at steady-state conditions (30 mA/cm^2^ constant current) and only showed performance loss measured as voltage increase after several minutes of continuous X-ray irradiation. We therefore conclude that beam damage was not relevant on the scale of our experiment, where the maximum exposure time was between 45 and 90 s for the imaged samples.

**Table 1:** XTM Setup and Parameters

element/method	description/parameter
5% white beam filter	20 mm of Sigradur and 75 μm of molybden
sample–scintillator distance	50 mm
scintillator	150 μm LuAG/Ce
macroscope	OptiquePeter, 4× magnification^29^
camera	GigaFRoST^28^
exposure	1 ms per projection, 1000 projections over 180°
projection images	size 2016 × 2016 pixels, pixel size 2.75 μm
phase filter	parameter: *E* = 21 keV, δ = 0.35 × 10^–^^7^, β = 1 × 10^–^^9^, propagation distance = 50 mm
tomographic reconstruction	Gridrec algorithm;^31^ image gray value scaling: mintiff=–1 × 10^–^^5^, maxtiff = 5 × 10^–^^5^, 16bit

### Image Data Analysis

#### Basic Processing

After reconstruction, the 3D images were rotated to have their features in the same straight orientation in all three spatial dimensions for all samples and then cropped to reduce the data size. Every scan was rigid-body-registered against the first *operando* scan of the corresponding sample, i.e., misalignment between the scans was corrected.

#### Machine Learning Segmentation of Delaminated Membrane Volume

The segmentation of the gas phase within the BPM is not trivial. The obtained gray value distributions for water, membrane, GDL carbon fibers, and gas phases overlap over almost their entire distribution and make threshold-based segmentation, e.g., after denoising or edge-enhancement, impossible. Also, time differential methods,^32^ e.g., image changes to some reference image, cannot be applied due to the strong and irregular deformation of the sample during acquisition. Machine learning (ML) segmentation employing a random forest classifier has proven to be a powerful segmentation technique in difficult cases.^34,35^ However, current implementations are defacto limited to 2D^34^ and 3D,^36^ while time-resolved (4D) data contains differential information valuable for segmentation like temporal gray value gradients indicating local changes of phase, e.g., gas to liquid.^35,37^ In this work, we extend the concept in ref (34) to create a pixelwise feature set by applying a series of 2D image filters and to train a random forest classifier to segment the images. For example, similar values of the Gaussian blur indicate coherent material phases, while strong gray-value gradients indicate phase boundaries. The classifier trained by manually labeling a small set of images effectively uses a combination of the pixel-wise features to attribute all pixels to one of the phases provided by the labels. We employ 4D image filters to harvest local gray value correlation in all three spatial dimensions including the temporal evolution. The employed filters and parameters are listed in Table 2. The method was implemented in Python and is made available (gitlab.psi.ch/fcsd_5422/pytrainseg). The image data was iteratively labeled and trained on 2D slices at all orientations and various time steps until a subjectively good segmentation was achieved.

**Table 2:** Employed Image Processing Parameters in Order of Application^a^

step	method	values
rotation	SciPy ndimage	manually and individually for each sample
rigid-body registration	SimpleElastix	slightly modified default parameters (gitlab.psi.ch/fcsd_5422/coely_tomcat_processing)
CL segmentation	scikit-image	Gaussian blur (3D): σ = 1 px, threshold = 15,000 (16 bit)
CL hole detection	cuCIM, CuPy	binary dilation (3D): ball kernel with radius 2 px, distance between initial planes: 5 px, difference threshold for hole detection: −0.5
4D ML segmentation	NumPy, scikit-image, SciPy ndimage, scikit-learn	image filters: Gaussian blur, difference of Gaussian blurs, gradient, Hessian matrix, first, last, mean, and minimum in time series, difference to first, last, and minimum image in time series. σ for blurs: 1, 3, 6 px. Derivative filters are applied for all σ. Scikit-learn random forest classifier: *n* = 300, seed = 42, maximal feature length

a Most processing steps employ the Python modules *joblib* and/or *dask* for parallelization.

#### CL Damage Detection

**Figure 2:**
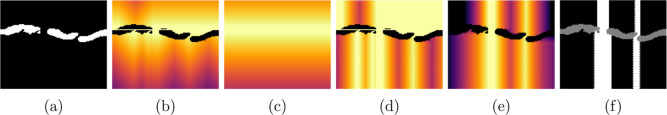
Illustration of the hole detection algorithm for a small region of interest. (a) Binary image showing broken CL, (b) dilation starting from the fine line with CL as an obstacle, (c) reference dilation, (d) difference, (e) average difference for all dilation repeats, (f) threshold for hole detection. The corresponding Supporting Video shows all dilation iterations.

Binary images of the respective anode and cathode CLs were obtained by first manually cropping the images to only include the respective CL, then slight denoising using a 3D Gaussian filter, and finally grayscale thresholding. Blender (blender.org) was employed to produce animated 3D visualizations. A custom procedure was developed to measure the damaged CL area. Projecting the segmented CL and measuring the empty area are insufficient to obtain reliable measurements. Visual inspection of the animated 3D image data reveals that broken CL pieces were not immediately detached, still covered the damaged area, and led to an underestimation of the damaged area with simple projection. A pseudocomputational fluid dynamics method was employed to obtain a more accurate measurement. The algorithm is illustrated in Figure 2. Based on the binary image of the CL (Figure 2a), the background was filled by iterative 3D morphological dilation initiated from a plane (fine line in Figure 2b) parallel to the CL, i.e., the plane grows thicker for each iteration, mimicking a diffusive process. The CL obstructs dilation, and the number of iterations required to fill the entire image was counted (Figure 2b). The same process was repeated without the CL (Figure 2c), and the difference was calculated (Figure 2d). The entire procedure was repeated by initiating the dilation at every height location of the image, and the average difference of all repeats was calculated (Figure 2e). By applying a suitable threshold (Table 2), all holes could be reliably detected, including when the direct path is obstructed (Figure 2f). The damaged area was finally calculated as the average of filled areas of the top and bottom planes (Figure 2f). The proposed method results in a reliable measurement of the damaged CL area with a slight overestimation (Figure 2f). All processing was performed using custom Python code (available at gitlab.psi.ch/fcsd_5422/coely_tomcat_processing), and the main parameters are listed in Table 2.

## Results

**Figure 3:**
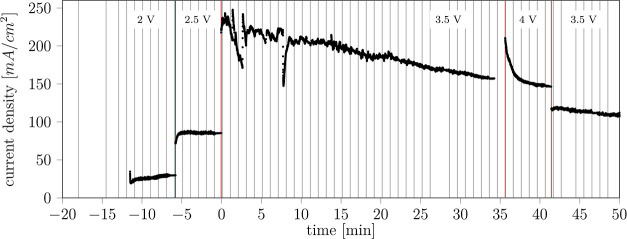
Electrochemical performance during image acquisition for sample 7. The sample was operated at constant voltages indicated by the values between the red bars. Vertical gray lines denote tomographic scans. *t* < 0 refers to preoperation conditioning.

A total of 10 cells were prepared and operated under variable electrochemical conditions while recording tomographic scans to explore a range of operation responses. Table 3 details the operating conditions. As an example, one sample (7) is discussed in detail throughout the Results section. Figure 3 shows a typical operation protocol. Sample 7 in Figure 3 was operated at a constant voltage of 3.5 V, and the resulting current density was recorded during 1 h of operation. A tomographic scan was taken every minute. All samples showed unstable performance despite nominal operation at a steady state of constant voltage or current (Figure S3).

**Table 3:** Description of the Applied Electrochemical Operating Conditions for All Tested Samples

sample	short description	value
1	constant current	30 mA/cm^2^
2	constant current for 45 min, then current ramp-up in 25 min	30, 200 mA/cm^2^ (ramp max.)
3	constant current	50 mA/cm^2^
4	constant current	50 mA/cm^2^
5	constant voltage	3 V
6	constant voltage	3.5 V
7	constant voltage	3.5 V
8	current ramp-up in 10 min	0–120 mA/cm^2^
9	current ramp-up in 10 min	0–200 mA/cm^2^
10	reference sample without applied voltage	n.a.

**Figure 4:**
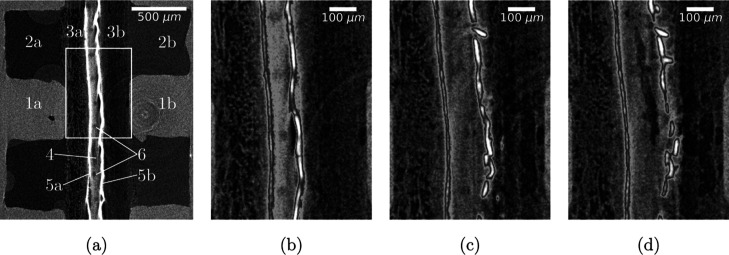
Sample 7: (a) tomographic slice before operation, cathode to the left. 1-polar plates, 2-flow field channels, 3-GDLs, 4-BPM, 5-CLs, 6-BPM fiber reinforcement. The white frame inscribes a region of interest showing the MEA (b) before operation and during operation after (c) 18.2 min and (d) 48.5 min. The bright CL was visually enhanced in (b–d) only for better readability using a modified Butterworth filter (eq S1 and Figure S4).

All figures that show selected time steps are additionally included as videos of the entire time series in the Supporting Information with the corresponding figure number. Figure 4a gives an example of an *operando* tomographic slice that allows a clear visual distinction of all components described in the experimental setup and Figure 1a. The CLs contain noble metals (Ag and Ir) with high X-ray attenuation and appear as bright lines. Figure 4b–d focuses on the MEA in a small region of interest. Substantial swelling of the membrane (Figure 4c) is observed together with the formation of a wide crack-like delaminated zone in the center of the membrane at late times (Figure 4d). Further, the anode CL disintegrates over time (Figure 4b–d). The two damage processes are discussed in detail in the following sections.

### Formation of Cavities in Membrane

**Figure 5:**
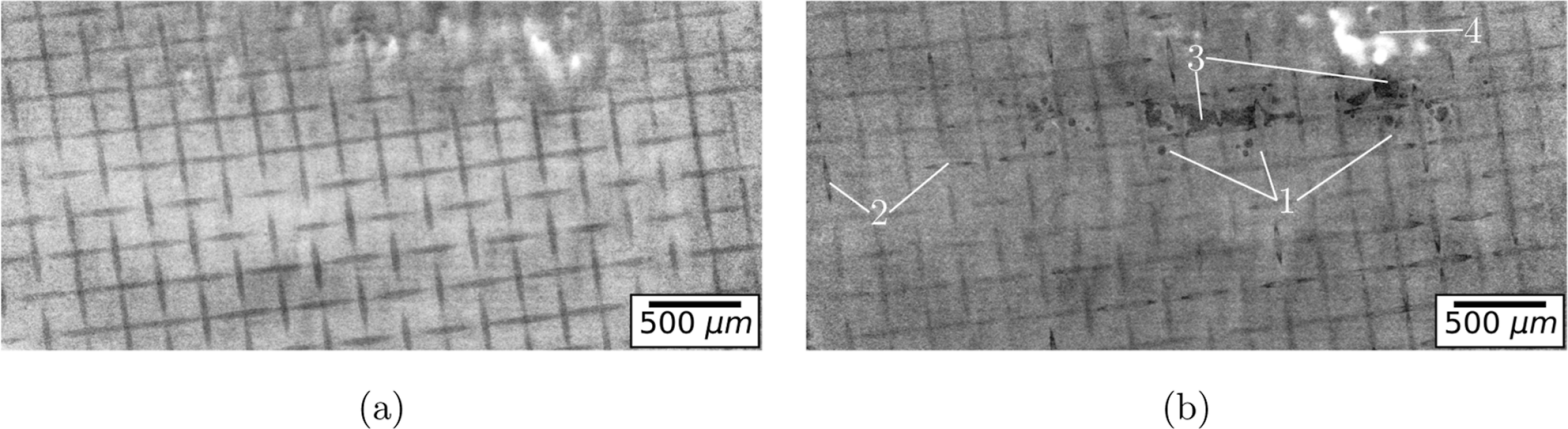
Tomographic slice through the membrane showing the different types of delamination (sample 7). (a) Before operation, (b) 38.7 min. 1-Bubble cavities, 2-fiber delamination, 3-areal delamination, 4-cut through CL due to bend in nonplanar membrane. Video in the Supporting Information.

Starting from the qualitative first observation in Figure 4, three types of delamination and cavity formation are observed in the tomographic images. Figure 5 shows a slice through the membrane for two selected time steps of sample 7 as (i) bubble cavities, (ii) fiber delamination, and (iii) areal delamination. The reinforcement weave made from PEEK fibers appears clearly as a dark grid pattern. Initially, small bubbles appear in the space between the fibers, while the ionomer also detaches from the fibers. These initial small cavities are not always stable and disappear in some cases. Then, the growing bubbles and fiber detachments merge to form larger areas of delaminated membrane; these bubbles can be observed for all samples and conditions. Finally, larger-scale delamination occurred, which however appeared only for the samples that were operated at higher current densities (above ≈100 mA/cm^2^, samples 2, 6, and 7.)

**Figure 6:**
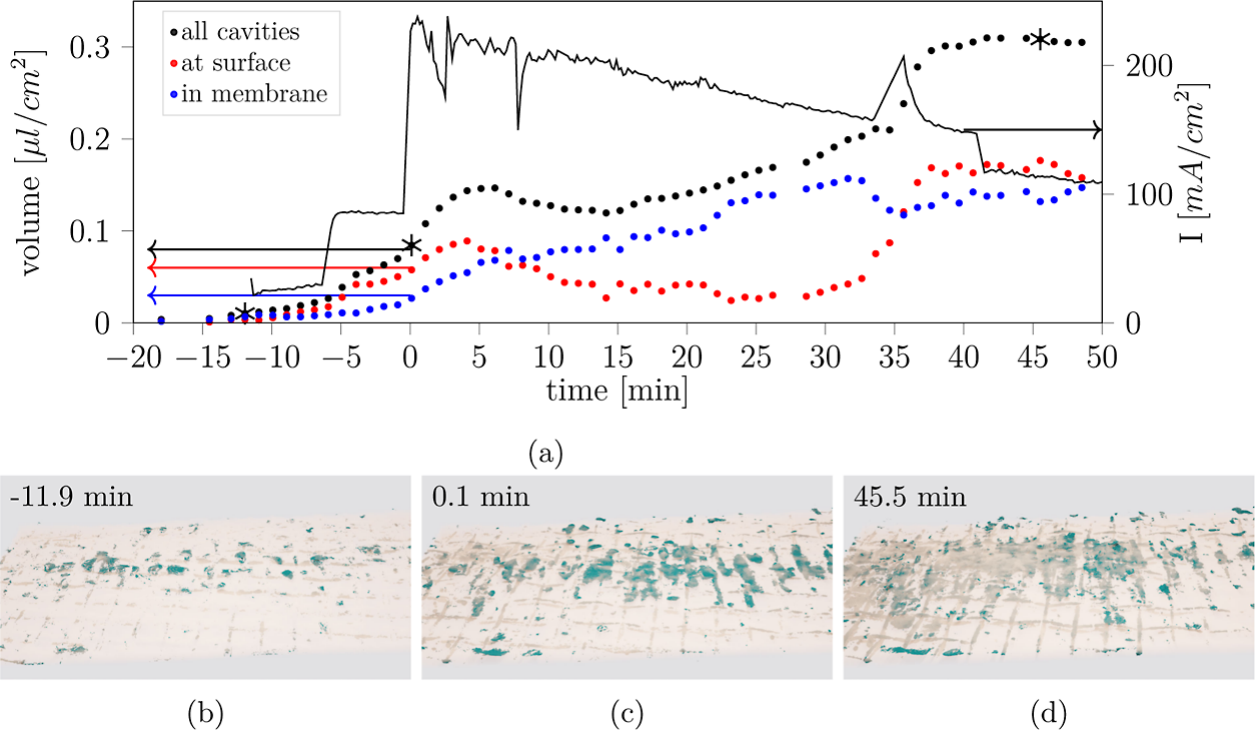
(a) Evolution of membrane cavities and current density for sample 7 operated at 3.5 V (for *t* > 0). (b–d) 3D visualizations of the segmented membrane marked with asterisk in (a). The cavities are displayed as solid green, while the membrane is kept in a semitransparent cream color. Surface cavities appear fully green, while cavities within the membrane are shaded.

**Figure 7:**
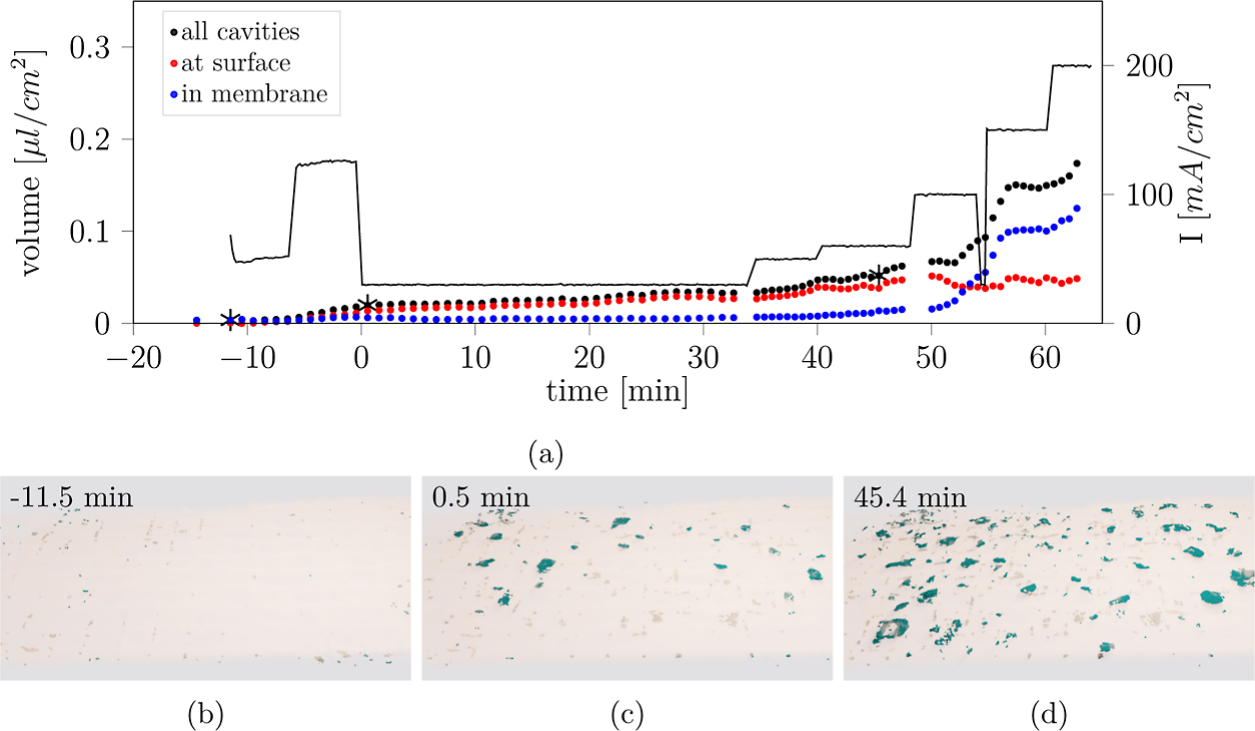
(a) Evolution of membrane cavities and current density for sample 2 operated at constant current (for *t* > 0). (b–d) 3D visualizations of the segmented membrane marked as asterisk in (a). The cavities are displayed as solid green, while the membrane is kept in a semitransparent cream color. Surface cavities appear fully green, while cavities within the membrane are shaded.

The samples 2, 6, and 7 are therefore examined in more detail. The 2D tomographic slices only give an incomplete picture of the affected volume within the membrane and are therefore unsuitable for quantification. Thus, the grayscale image data was segmented using the custom 4D ML segmentation method assigning each pixel (*x*, *y*, *z*, time) to either membrane, gas within the membrane, or other (CL, GDL, water, etc.) to quantify the delamination volume. Sample 6 showed large fractions of delaminated areas already before operation (Figure S5) not present in any other sample, which indicates pre-existing damage of the membrane and is disregarded for the following analysis of membrane delamination. This initial membrane delamination in sample 6 leads to much larger membrane cavities from the beginning of operation compared with those in the other samples (Figures 6, 7 and S5). Figures 6 and 7 show the time-resolved 3D visualization of the gas cavities in samples 2 and 7. Some small cavities at the anode membrane–CL interface and at the reinforcement fibers were present in all cases already before operation, indicating imperfect membrane ionomer adhesion to the fiber material.

Samples 7 and 2 were operated under contrasting conditions (Table 3): for sample 7, the current density was high from the start (*I* > 200 mA/cm^2^, Figure 6a). Sample 2 (Figure 7a) was operated at low current density (30 mA/cm^2^) for 45 min followed by a ramp-up to a similarly high level (≈200 mA/cm^2^).

For sample 7, delamination at fibers is visible early on (Figure 6b). Larger delaminated areas appear late (Figure 6d), triggered by the short interval of applying 4 V, which quickly raised the current density. In 3D, it becomes clear that the delaminated fibers form a continuous network of cavities guided by the weave pattern. Inspection of the segmented 3D image data also reveals an additional distinction of membrane cavities initially not susceptible in the 2D grayscale slices. One part of the cavities is located at the center of the membrane and a second part at the membrane surface toward the anode CL. To computationally distinguish the cavity types, the segmented anode CL was dilated (sphere, radius 3 px) as a reference. Any separated cavity overlapping with the dilated CL was assigned to the “surface cavities”, while the remaining cavities belong to “membrane cavities”. Figure 6a contains the different cavity volumes over time compared with the current density. The total cavity volume within the membrane generally increases with time. The “surface cavity” volume decreases after an initial rise. The decrease can be correlated to the appearance of surface defects in the anode CL at about 5 min by comparing the temporal evolution of the segmented membrane with that of the anode CL (Figure S6, same frames of supporting Videos S8 and S10). The ML segmentation algorithm attributes the cavities at the membrane surface to the background after the removal of the covering CL, virtually making them disappear (Figure S6). As a reasonable physical explanation, the cavities between the membrane and CL have transformed into a proper hole in the CL. The same behavior is observed for sample 2 at *t* ≈ 50 min, where a decrease of surface cavity volume (Figure 7a) corresponds to a sharp increase of damaged anode CL area (Figure S7b). In the same period, two sharp and reversible drops (Figure 6a: *t* ≈ 2 min, *t* ≈ 8 min) appear in the recorded current density. However, we could not observe any clear correlation of the drops in the current density to structural processes captured by imaging. The steep rise of the “surface cavity” volume at about 35 min (Figure 6a) corresponds to the formation of the large cavity inside the membrane (Figure 4d), which appears to be connected to the detected cavity volume in the proximity of the CL and therefore counted as “surface cavity” instead of “membrane cavity”.

Sample 2 shows different behavior (Figure 7). During periods of low current density (<60 mA/cm^2^), nearly all cavity volume is located at the surface. Raising the current density above 100 mA/cm^2^ triggered the formation of cavities within the membrane itself. We attribute the occasional decrease of the total cavity volume to the actual disappearance of cavities and to uncertainties of the image segmentation. The cavities can also be attributed differently (surface vs within membrane) due to the changes in shape and spatial extension. We learn from both samples a general trend: for low current densities (<100 mA/cm^2^), gas cavities were formed primarily at the membrane surface, while the larger part of the formed gas cavities that were located inside the membrane appeared at higher current densities. However, we hesitate to suggest a universal correlation between electrochemical operation and cavity volumes due to the limited available data.

### Anode CL Damage

**Figure 8:**
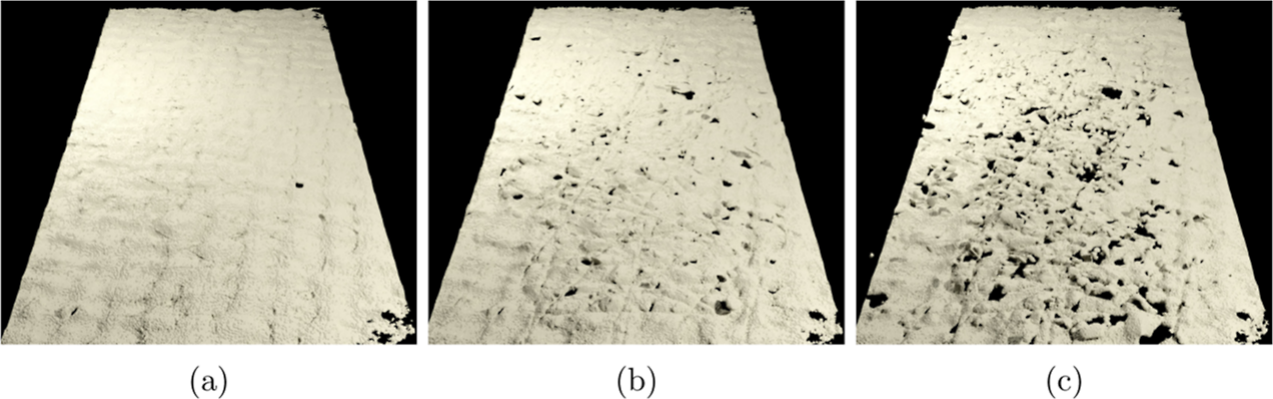
3D visualization of the segmented anode CL seen from the anode in sample 7 (a) just mounted and after (b) 7.1 min and (c) 47.5 min of operation. The displayed width of the CL is 2 mm. Video in the Supporting Information (Video S10). All samples in Figures S7 and S8.

While delamination at the BPM junction is observed only for the samples operated at high current density, all samples show a clear damage of the anode CL during operation. The image series in Figures 4b–d and 8 show the subsequent breakage and detachment of pieces in the size of tens of micrometers that lead to a notable disintegration of the anode CL. The straight impression marks are caused by the membrane swelling and pressing the CL against the fibrous GDL.

The same qualitative behavior is observed for all samples (Figure S8) except for reference sample 10 with no voltage applied, where the CL stays completely intact (Figure S8a,b and Video S11). Starting from an intact layer (Figure 8a), an increasing number of pieces are broken off and lifted out of the CL (Figure 8b,c). The damage is distributed over the entire active area. All broken pieces are lifted toward the anode GDL away from the underlying membrane. Also, many holes have a crater-like shape pointing away from the membrane (Figure 8).

**Figure 9:**
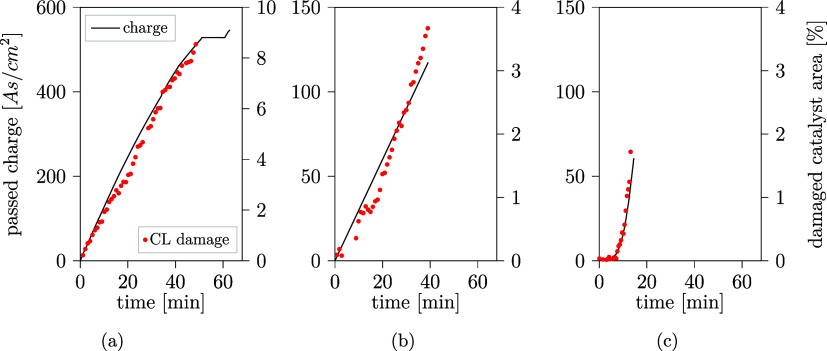
Passed charge (black) and damaged anode catalyst area (red dots) plotted against time for operation at (a) sample 7, constant voltage at 3.5 V, (b) sample 4, constant current density 50 mA/cm^2^ and (c) sample 9, current ramp-up from 0 to 200 mA/cm^2^ in 15 min. All samples in Figure S7.

The observations in Figures 4–8 point toward the formation of a gaseous species within the membrane and at the anode CL. We quantify the CL damage in relation to the electrochemical operation. For three exemplary samples, at different operating conditions, the cumulative passed charge (current integrated over time) and the fraction of damaged CL are plotted against time in Figure 9. All remaining samples are shown in Figure S7. Despite the short operation time of around 1 h for all samples, large fractions of up to almost 15% (sample 2) of the anode CL were damaged. Both, the damaged area and charge are calibrated, i.e., set to zero, to the end of conditioning and the start of the actual operation. Despite the effectively very different applied electrochemical operating conditions (Table 3), the evolution of charge and CL damage match remarkably for all samples.

**Figure 10:**
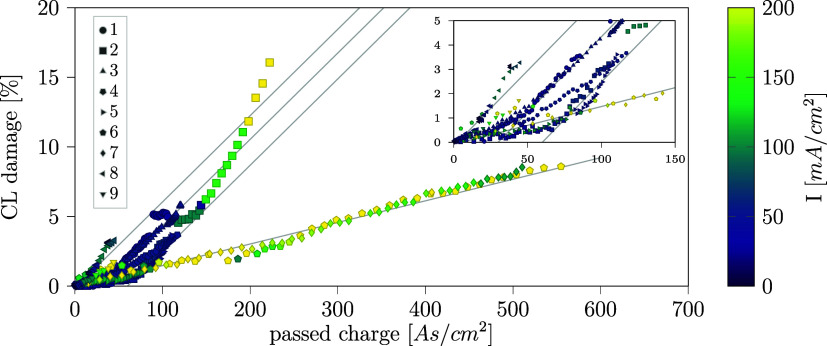
Anode CL damage versus passed charge for all samples. Inset: data for low charge less than 150 A s/cm^2^.

Given the apparent correlation of charge and anode CL damage in Figures 9 and S7, the CL damage is plotted against charges in Figure 10 for all samples at every XTM measurement point, and the current density is indicated with a color code. Two types of proportionality can be detected as two slopes of linear correlation. While all samples follow the same flat slope (0.0155%/(A s/cm^2^)) at first for low charge, only the samples operated at elevated (above 150 mA/cm^2^) current density maintain this correlation (6, 7). All other samples operated at lower current density transition to the same steeper slope between 10 and 100 A s/cm^2^ of passed charge (Figure 10). We note that the gradient of the steep slope is four times that of the flat slope, i.e., the CL shows a four times higher damage rate with larger damaged area per passed charge.

### Further Observations

From the image evaluation, the following secondary observations can be made: (i) the cathode CL remains undamaged and shows no apparent changes during operation; (ii) large gas bubbles in the size of tens of micrometers can be observed in the water-filled anode GDL, and (iii) some pore space of the cathode GDL is filled with water. Observations (ii) and (iii) are relevant for the water management in the cell and will not be discussed here but later on in a separate analysis of the water transport and sorption characteristic of the forward-bias BPM CO2ELY cell.

## Discussion

The imaging data provide valuable insights into the discussion of degradation mechanisms in forward-bias BPM-CO2ELY. We can exclude cathode flooding and carbonate salt clogging as degradation processes, as observed for AEM CO_2_ electrolysis (AEM-CO2ELY). The pore space of the cathode GDL remains open with only a little space filled by liquid water. Solid deposits like salt are not observed in significant quantity.

The imaging data, however, show gas formation leading to substantial damages at the AEL–CEL junction in the BPM and at the anode CL. We hypothesize that the gas is CO_2_ originating from the reactions of the carbonate charge carriers at the BPM junction. The fast chemical reactions of CO_2_ at the cathode (equation) replace an important portion of the negative OH^–^ charge carriers in the AEL with (bi)carbonates. A CO_2_ concentration gradient is built up from the BPM junction to both electrodes once the carbonate ions are transformed to neutral CO_2_. The CO_2_ transport mechanism shifts from electric-field-dominated electro-osmotic drag acting on the carbonate ions (Nernst–Planck equation) to Fickian diffusion of CO_2_ (Fick’s first law). The consequence of the CO_2_ concentration gradient is diffusive flux of CO_2_ toward both electrodes, which we attempt to simulate with a diffusive 1D-continuum model (Supporting Information, Section S8). Solving the model for the steady state, we obtain a pressure of 841 kPa for the gas phase at the BPM junction at a current density of 100 mA/cm^2^. The obtained pressure is, based on available elastic modulus data,^38^ sufficient to compress the cathode and anode GDLs with the inflating the membrane.

The ionomer–PEEK interfaces at the PEEK reinforcement fibers at the junction of the BPM act as weak points for the first gas formation within the membrane. The delaminated fibers also form a connected network of cavities that can allow for in-plane gas transport. Eventually, increased delamination at the BPM junction is observed, as presented in the Results section. Gas-induced cavities are not only observed at the BPM junction but also at the interface of membrane and anode CL. The image data suggests that the threshold to form gaseous CO_2_ and to build up sufficient pressure to expand the gas cavities is different for the BPM junction and membrane–CL interface. Higher current, and therefore, higher CO_2_ concentration, is required to delaminate the BPM at the junction than forming gas pockets between the membrane and CL. The formation of cavities at the given interfaces can be dependent on multiple parameters like ionomer composition, interface adhesion, hydration, and pre-existing pores. Further studies are required to determine the importance of material interface properties and how they influence the delamination at the different locations of the membrane under differing operating conditions.

The cavities between the membrane and anode CL are a precursor to the observed anode CL damage. Given sufficient CO_2_ accumulation and therefore pressure, the gaseous CO_2_ breaks through the CL by punching holes. The set time resolution of the image acquisition, however, is insufficient to capture the actual breaking event of the CL. CO_2_ flux across the mainly liquid-filled anode CL leads to a considerable pressure drop of 188 kPa at 100 mA/cm^2^ in the model due to the low porosity and high tortuosity (Table S1) of the CL. Based on our model, a CO_2_ crossover of 0.296 μmol/cm^2^ s (Table S2) to the anode is predicted. The CO_2_ crossover is not fully eliminated despite the strong reduction by employing a BPM and is in the same order of magnitude as experimentally measured (0.7 μL/cm^2^ s ≈ 0.03 μmol/cm^2^ s).^4^

In the model, the CO_2_ concentration at the interface between BPM and anode CL is above the solubility limit of 19.5 mol/m^3^ at 45 °C and ambient pressure.^39^ The tabulated solubility limit is equivalent to 52 kPa CO_2_ pressure in our model. Experimentally, the process unfolds in a way not directly captured by the model. The diffusivity and solubility of CO_2_ are too low to maintain the CO_2_ flux across the anode CL as dissolved species. Gas cavities form, and the transport across the anode CL happens in the gaseous form. The capillary breakthrough pressure for gas percolation through the porous anode CL is in the range of hundreds of kPa as approximated by the Young–Laplace law using measured pore radius distribution peaking at around 200 nm.^40^ The pressure of the formed gas bubbles is therefore insufficient for gaseous percolation of the CL. CO_2_ is trapped between the membrane and anode CL until the built-up pressure is sufficient to break the CL (Figure 8). The damaged CL area was extracted from the image data with an appropriate processing method (Figure 2). A strong correlation is found between the damaged CL area and the passed charge for all samples (Figure 10) for the first hour of operation. Qualitatively, there appears to be a typical size for CL holes in the range 10 μm, which are evenly distributed over the active area. Damaged areas become electrochemically inactive across the MEA, so that CO_2_ formation seizes at the damaged site and CO_2_ accumulation shifts to a different location. The assumption of, on average, equal CO_2_ accumulation and pressure before breaking a hole into the CL would explain the strong correlation between the charge and damaged area. Simply put, each amount of charge carrier produces a certain number of CO_2_ molecules. If sufficient CO_2_ molecules have accumulated at a site, then the CL breaks with a typical hole size. Similar to the formation of cavities in and on the membrane, the quantitative anode CL damage rate will depend on a multitude of material properties including thickness, stiffness, composition, and adhesion to the membrane. In first-order approximation, we extrapolate the lower damage rate in Figure 10 and predict a 100% damaged CL area after operating the electrolyzer for 9 h at 200 mA/cm^2^. A more sophisticated electrochemical model coupled with gas formation and mechanical stresses would be required for a detailed assessment, but this is out of scope in this work. The transition to a higher damage per charge rate primarily for lower current density (Figure 10) is a curious observation. A possible explanation is that the charge carrier distribution in the anionic part of the BPM shifts from carbonate ions to OH^–^ at higher current density resulting in reduced CO_2_ crossover per transported charge. Consequently, the damage vs charge rate would decrease. However, modeling of AEM-CO2ELY suggests the transition from carbonate to OH^–^ only at a higher current density (500 mA/cm^2^)^41^ compared to the experimentally observed split of damage rates here at about 150 mA/cm^2^. Since only two samples show a lower damage rate, we suggest further experimental research to address the relation of current density and damage rate. Since synchrotron beamtime is scarce, an alternative study could be performed as a *post-mortem* analysis comparing the membrane condition before and after operation at different current densities and accumulated charge.

Both membrane delamination and anode CL damage also occur when employing a typical Ti PTL and are not due to the presence of soft carbon paper at the anode (Supporting Information, Section S7 and Figure S10). We do, however, expect that reducing the PTL surface porosity, e.g., with an MPL, will provide support to the anode CL and reduce degradation.

While our proposed mechanism is physically well reasonable and in line with our experimental observation with our experimental observation of reduced but not eliminated CO_2_ crossover for BPM-COELY,^4^ we cannot ultimately prove with our methods that the observed cavities are actually filled with CO_2_. However, since the imaging data clearly shows a gas, we can test other gaseous species potentially present in our system, namely, vacuum, H_2_, CO, water vapor, some membrane decomposition product, and O_2_. Vacuum can be rejected because a positive pressure difference in the environment is necessary to form the cavities. H_2_ and CO can only be formed at the cathode CL and are quickly removed by the cathode gas stream and, in the case of H_2_, only show low crossover rates for even thinner membranes and higher current density.^42,43^ Water vapor can be excluded because the pressure needed to compress the GDL, delaminate the membrane, and perforate the CL would lead to vapor condensation at our operating conditions (9.6 kPa vapor saturation pressure at 45°). Liquid water is much denser than vapor and can clearly be distinguished by XTM.^44^ There are no signs of polymer decomposition in the image data but rather a clear cracking pattern.^45^ To the best of our knowledge, there is no low-temperature polymer decomposition process that would produce gases at a sufficient pressure to inflict the observed structure changes, leaving only O_2_. Oxygen production in the gas phase (vapor electrolysis) is limited,^46^ and we do not expect sufficient oxygen pressure build up to break the anode CL. Furthermore, oxygen evolution cannot explain the membrane delamination at the junction. CL damage is also not observed in water electrolysis.^22^ However, better adhesion of CL to the employed Nafion212 in ref (22) compared to our material combination might resist similar anode CL degradation. To ultimately rule out oxygen-induced anode CL degradation, it is necessary to test several membrane–catalyst combinations not only in CO_2_ coelectrolysis but also in water electrolysis eliminating any CO_2_ source, which was out of scope of this work. We do not currently see an experimental method that would otherwise allow local gas analysis on top of the spatial and temporal resolution achievable by XTM. No damage occurs at the cathode CL because CO_2_ is a reactant and can also easily migrate through the gas-open porous media if in excess or be consumed in the cathode reaction. Considering the qualitative match of our experimental observations with the physical process discussed above, we see our hypothesis of CO_2_ gas-induced degradation as the most reasonable explanation for the observed structural dynamics.

Despite the clear degradation processes, we do not observe an obvious effect on the electrochemical performance of the CO_2_ electrolysis. Only slight performance decrease or even improving performance over time (Figure S3) is observed on the scale of tens of minutes to 1 h. Typical degradation trends, e.g., rising voltage at constant current and decreasing current at constant voltage, could not be correlated to the observed damages. On the contrary, the samples show fast reversible instabilities (<1 min) that are much faster than the continuous degradation of the anode CL. In other terms, the CO2ELY performance is too unreliable to ascertain a performance degradation due to the observed damages.^47,48^ Other effects on a short time scale might effectively mask the continuous damage of CL and the membrane. First achieving a more stable electrochemical operation in general and then using a higher temporal imaging resolution could reveal more direct relation between electrochemistry and structural dynamics in the future. Gas bubbles in the liquid electrolyte are reported to lead to an unstable CO2ELY operation on short time scale,^19^ as well as gas bubbles in general electrochemical cells.^49^ The structural dynamics observed here as membrane (solid electrolyte) cavities, gas bubbles in the anode GDL (Figure 4b–d), and anode CL breakage may show a similar dynamic effect on the stability. The availability of water to the reaction (equation) at the gas-fed cathode,^12^ surface poisoning of the catalyst, and the hypothesized electrocatalytic behavior of the ionomer^6^ used in the catalyst ink or lack of cations might also contribute to the performance instability.

## Conclusions

CO_2_ electrolysis employing a BPM in forward bias (BPM-CO2ELY) is a promising method to address issues, in particular, salt precipitation and CO_2_ crossover, affecting pure alkaline zero-gap electrolyzer with an AEM (AEM-CO2ELY), but still suffers from poor stability. We employ time-resolved *operando* XTM to study degradation in BPM-CO2ELY. Flooding and salt precipitation known in AEM-CO2ELY^3,11−13^ are not observed. However, different degradation processes related to the formation of gaseous CO_2_ are identified.

Our study highlights the importance of addressing the CO_2_ transport mechanisms as an important topic of research in CO2ELY in general and for BPM-CO2ELY in particular. While a BPM greatly reduces the CO_2_ crossover to the anode as carbonate ions, carbonate recombination at the BPM junction introduces a new issue. CO_2_ formed at the junction of the BPM accumulates and forms gas bubbles that damage the anode CL and the membrane itself.

Further investigation is needed to fully understand and further optimize the CO_2_ pathways in BPM-CO2ELY since the structural damage will inevitably lead to performance loss. However, the structural damages observed in this study do not appear to be the main driver of the fast performance degradation reported for BPM-CO2ELY.^4,14^ There appears to be at least one other, currently unknown, process that affects the performance more quickly and more effectively. There is still potential for BPM-CO2ELY, considering that currently available BPMs are not optimized for forward-bias CO2ELY. Modifying the BPM, e.g., the composition of the layers^8^ and the junction,^50^ as a way to enhance CO_2_ back-diffusion toward the cathode should not only solve the degradation described in this study but also improve the CO_2_ utilization for electrochemical reduction. Currently available BPMs also show higher Ohmic losses than that in purely anion or cation conducting membranes^51,52^ and need to be improved for energy efficient application.

## Supporting Information Available

Animated time series of Figure S2b (AVI)
Animated time series of Figure S2c (AVI)
Animated time series of Figure S2d (AVI)
Animated time series of Figure S4a (AVI)
Animated time series of Figure S4b–d (AVI)
Animated time series of Figure S5 (AVI)
Animated time series of Figure S6b–d (AVI)
Animated time series of Figure S7b–d (AVI)
Animated time series of Figure S8 (AVI)
Animated time series of Figure S5b,c (AVI)
Animated time series of Figure S8a,b (AVI)
Referenced supporting figures, additional details of the experimental setup, recorded electrochemical data of all samples, image filter applied, control experiment employing a titanium PTL at the anode and the corresponding results, and derivation and application of the 1D diffusive model of CO_2_ transport within the BPM (PDF)

## Supplementary Material

ae3c02882_si_001.avi

ae3c02882_si_002.avi

ae3c02882_si_003.avi

ae3c02882_si_004.avi

ae3c02882_si_005.avi

ae3c02882_si_006.avi

ae3c02882_si_007.avi

ae3c02882_si_008.avi

ae3c02882_si_009.avi

ae3c02882_si_010.avi

ae3c02882_si_011.avi
