# Supplementary material for: Gas-Induced Structural Damages in Forward-Bias Bipolar Membrane CO2 Electrolysis Studied by Fast X-ray Tomography
Source: ACS Appl Energy Mater. 2024 Apr 19;7(9):3590–601. doi: 10.1021/acsaem.3c02882 (PMC11094683; doi:10.1021/acsaem.3c02882)
Supplement: Supplementary file 12 — ae3c02882_si_012.pdf [file ae3c02882_si_012.pdf]

Supporting Information to Gas-induced structural damages in bipolar membrane forward bias CO_2 electrolysis studied by fast X-ray tomography

Robert Fischer[1], Matthieu A. Dessiex[1,2], Federica Marone[3], and Felix N. Büchi[1,*]

[1]Electrochemistry Laboratory, Paul Scherrer Institut, 5232 Villigen PSI, Switzerland
[2]Laboratory of Renewable Energy Science and Engineering, Ecole Polytechnique Fédérale de Lausanne (EPFL), 1015 Lausanne, Switzerland
[3]Swiss Light Source, Paul Scherrer Institut, 5232 Villigen PSI, Switzerland
[*]felix.buechi@psi.ch

S1 Cell setup schematics

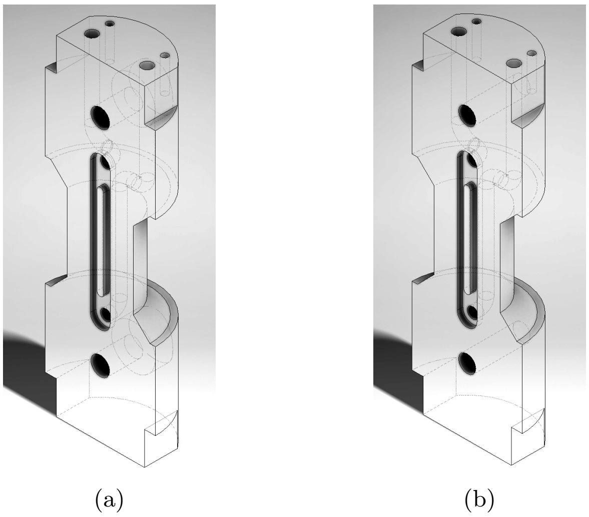

Figure S1: Drawings of the polar plates displaying the internal routing of the gas/water streams to the integrated flow field. Symmetric except for the screw thread. a) cathode, b) anode

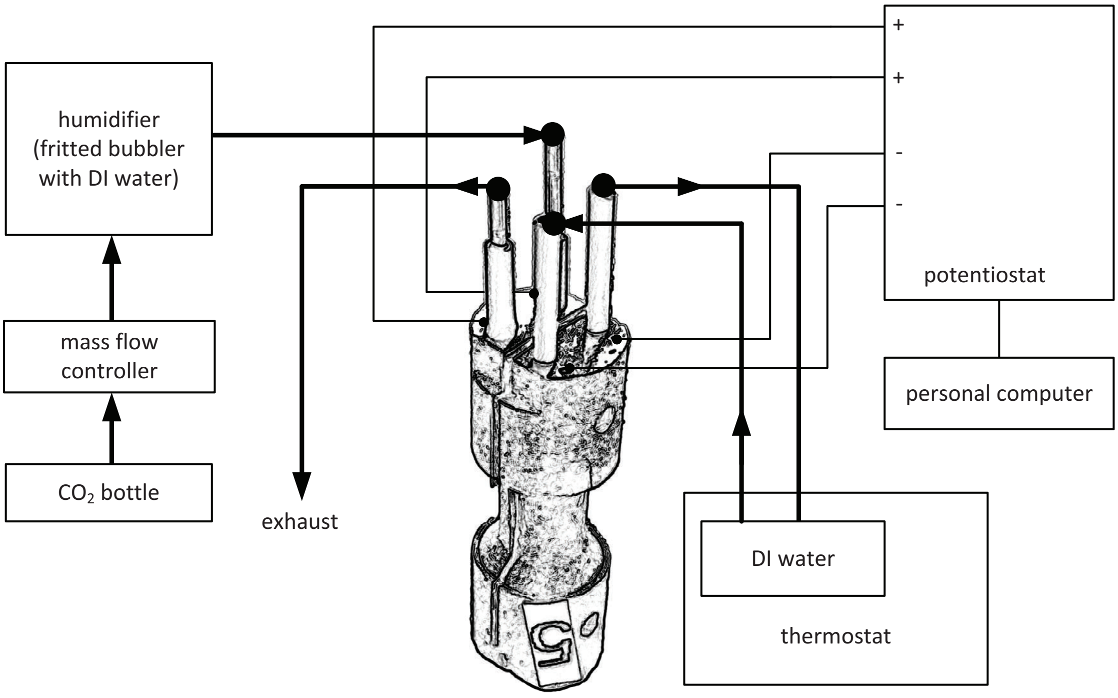

Figure S2: Schematics of the miniature co-electrolysis cell (stylized photography) with outlined tubing to water (anode), CO_2 (cathode) feed and electric connections to the potentiostat

S2 Electrochemical performance

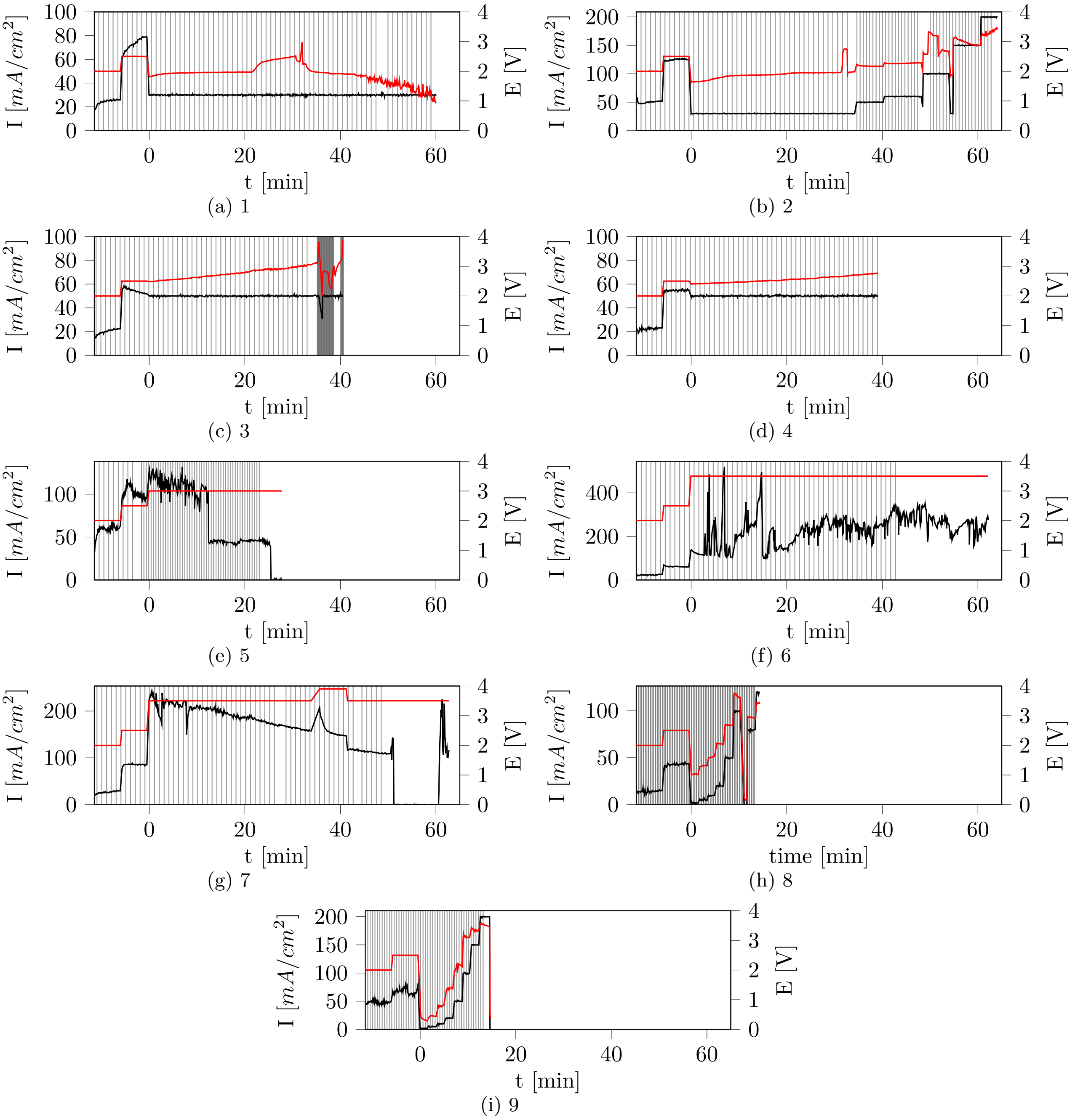

Figure S3: Current density (I, black) and voltage (E, red) over time (t) for all tested samples. Vertical gray lines denote tomographic scans. t=0 corresponds to the end of the conditioning step.

S3 Catalyst layer image filter

The metallic catalyst layers appear very bright in XTM due to their high X-ray absorption. In order to darken the corresponding image pixels for better readability and highlight the catalyst layer edges, the grayvalue $G(x, y)$ of a pixel at position x, y is reduced to $\hat{G}(x, y)$ using equation S1.

$$\hat{G}(x,y) = G(x,y)\left(1 - s + \frac{s}{1 + \left(\frac{G(x,y)^2}{g_c^2}\right)^n}\right) \tag{S1}$$

The image filter is inspired by the Butterworth Lowpass filter, but applied directly on the real space instead of the Fourier space. Bright pixels are mostly affected, while grayvalues way below the critical grayvalue g_c remain unchanged. n controls the roll-off and s is a value between 0 and 1 describing the strength of the filter. Values are manually chosen as $g_c = 14500$, $n = 20$ and $s = 0.265$.

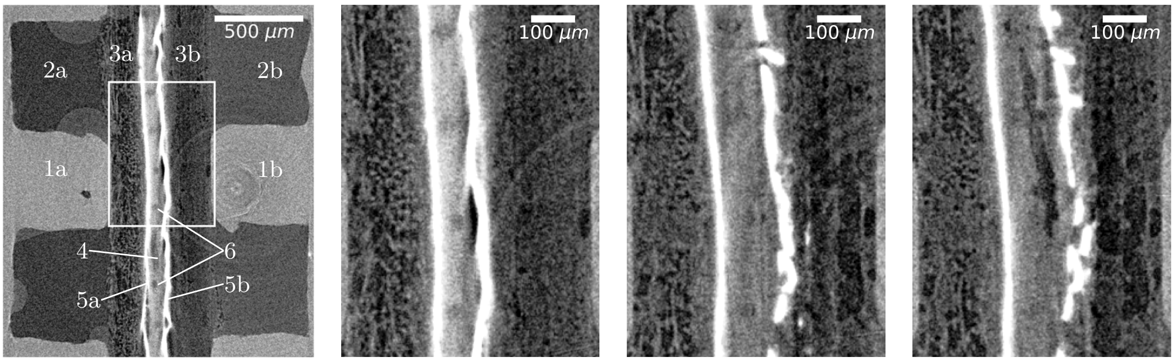

(a) (b) (c) (d)

Figure S4: Figure 4 in main text without filter.

S4 Gas cavities in membrane

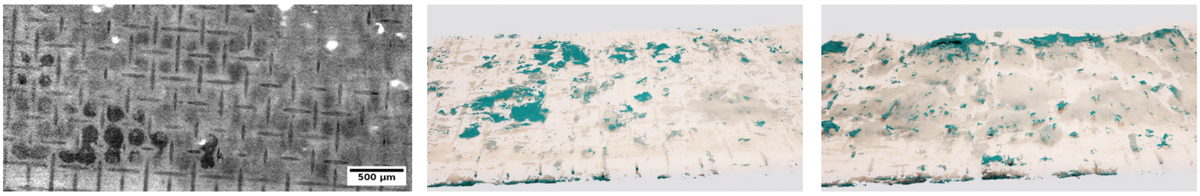

(a) (b) (c)

Figure S5: Cavities in BPM for sample 6. a) tomographic slice for the state before operation. 3D visualization of segmented cavity b) before operation and c) after operation. b),c) available as videos

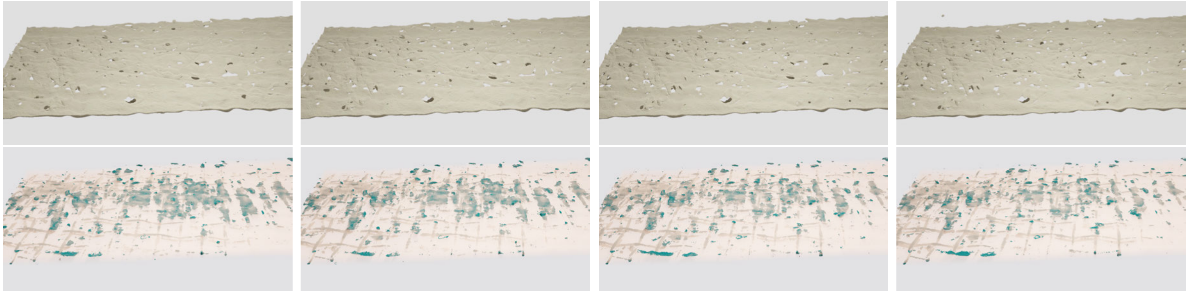

(a) 6.1min (b) 8.1min (c) 10.1min (d) 12.1min

Figure S6: Sample 7: Visualization of segmented anode catalyst layer (top row) and membrane cavities (green, bottom) for the period of decreasing ”surface cavity” volume (≈4-15min) in figure 6a of the main text.

S5 Correlation anode CL damage and charge

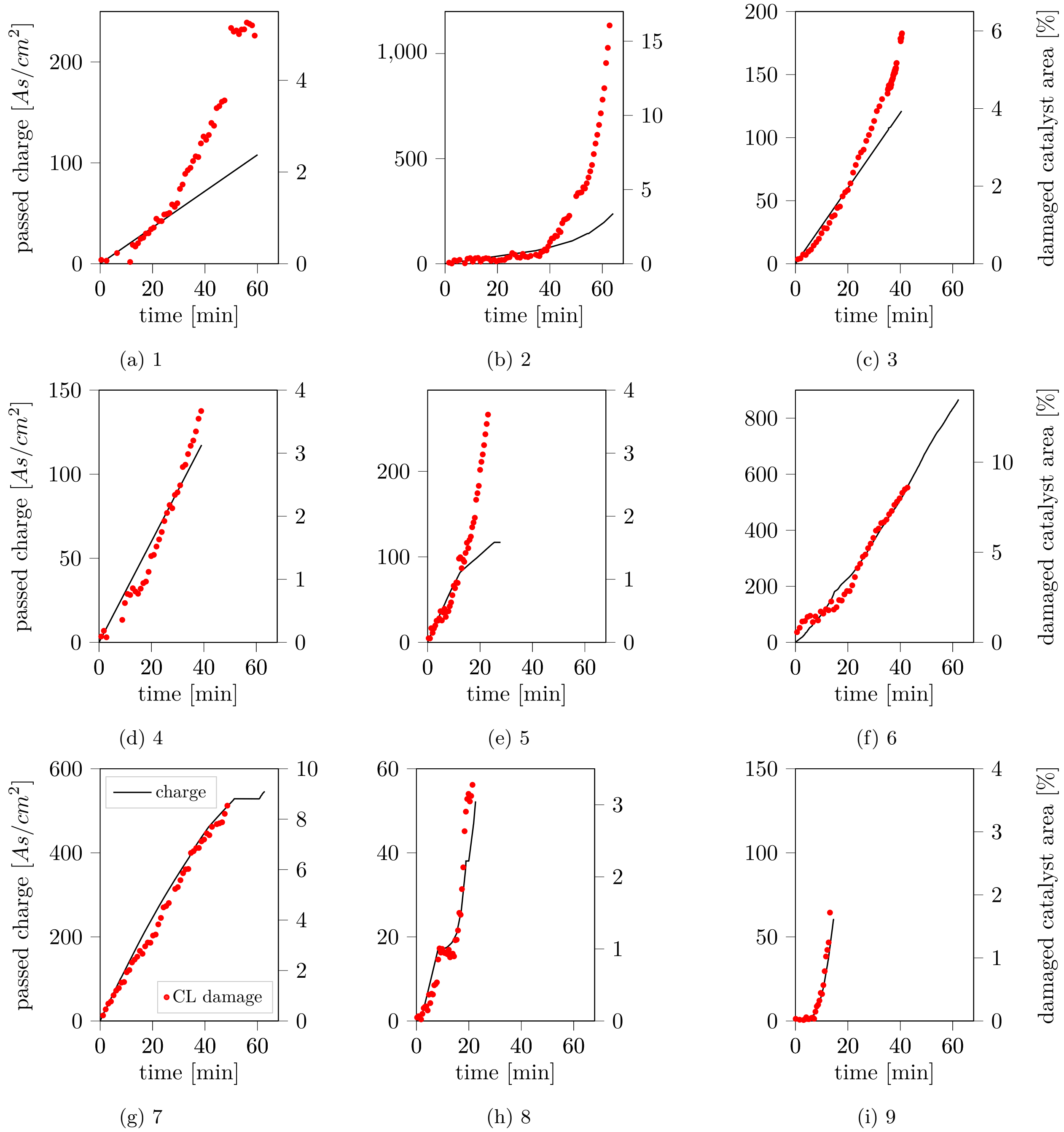

Figure S7: Charge (black) and damaged anode catalyst area (red) over time for all tested samples.

S6 Anode catalyst layer visualization

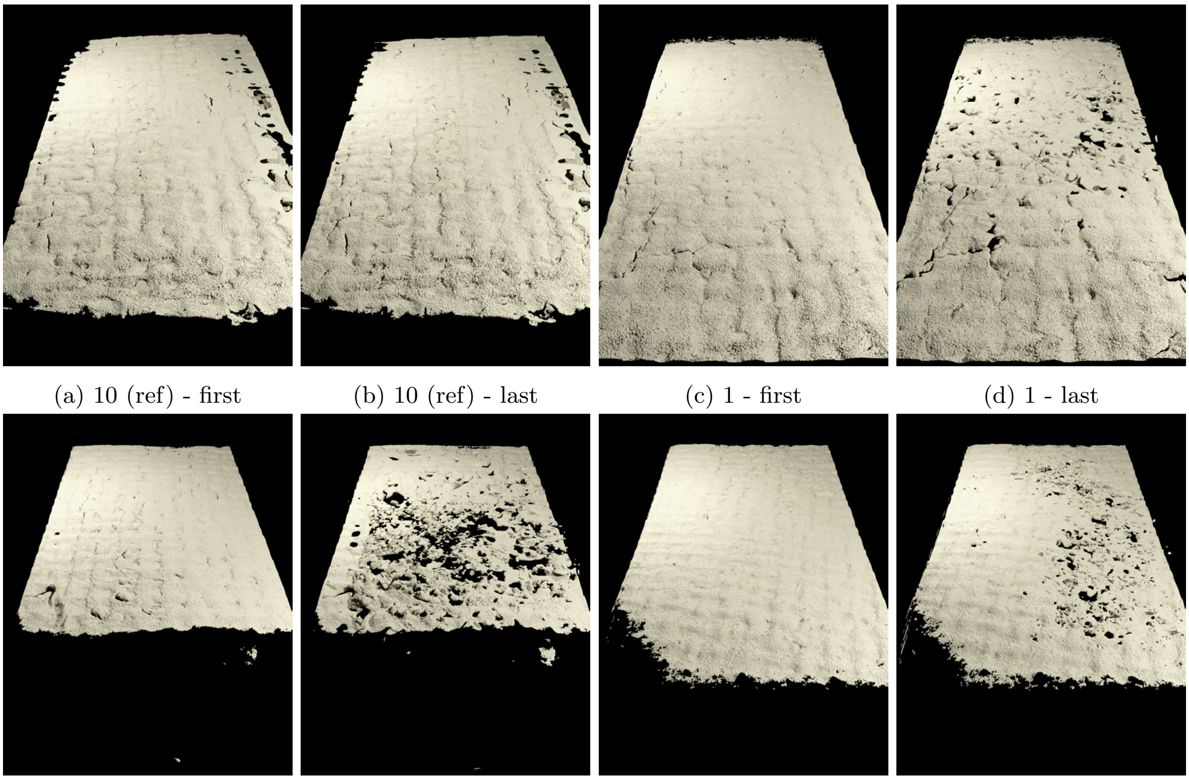

(a) 10 (ref) - first (b) 10 (ref) - last (c) 1 - first (d) 1 - last

(e) 2 - first (f) 2 - last (g) 3 - first (h) 3 - last

Figure S8: 3D-Visualization of the segmented anode catalyst layer at beginning (first frame) and end of operation (last frame) for all samples. We note that the CL was not always perfectly aligned with the field of view during image acquisition. In samples 10 (ref) and 8 (figure S9), the membrane is not flat resulting in a cropped region of interest containing parts of the cathode CL (right edge of images)

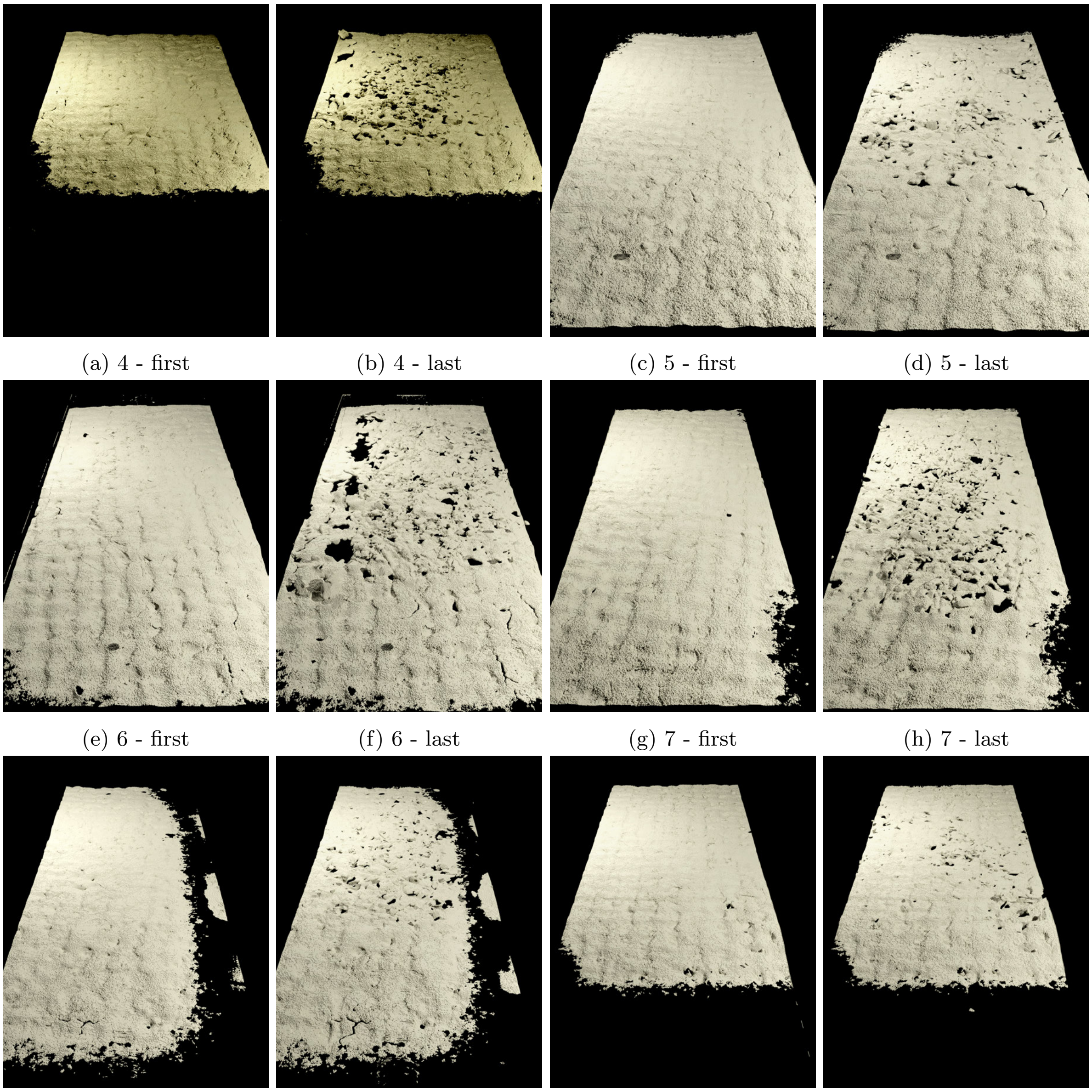

(a) 4 - first (b) 4 - last (c) 5 - first (d) 5 - last
(e) 6 - first (f) 6 - last (g) 7 - first (h) 7 - last
(i) 8 - first (j) 8 - last (k) 9 - first (l) 9 - last

Figure S9: Visualization of anode CL continued.

S7 Catalyst layer damage for a cell with Ti porous transport layer at anode

To test the applicability of our result to the typical co-electrolysis design, the experiment was repeated qualitatively with a Titanium porous transport layer (Ti PTL) at the anode using a tube X-ray cone beam CT scanner (Phoenix nanotm m, General Electric, Germany). The experimental setup is the same as for time-resolved XTM at the synchrotron except for the replacement of the carbon paper GDLs as anode PTL with two stacked 0.25mm thick Ti felts (2GDL10-0.25, Bekaert, Zwevegem, Belgium) and a thicker (0.47mm, FC-FKM 200, Freudenberg) ice cube gasket. The X-ray tube was set to 80kV acceleration voltage and 230μA. A 0.1mm Cu filter was placed into the beam to filter out low energy photons and reduce metal artifacts. 1000 radiographic projections were recorded equally spaced over one full rotation of 360°. Each projection is the average of three frames at the same angle with an exposure time of 0.5s each. The acquisition of one frame was skipped while the stage is moving to the next angular position. The sample was placed at a distance of 12mm to the X-ray source while the flat panel detector had a distance of 400mm to the source capturing the entire width of the cell in the field of view and resulting in a voxel size of 3μm. Tomographic reconstruction was performed using the implementation of the Feldkamp-algorithm for cone beams in the commercial software datos|rec (Waygate Technologies, Bake Hughes, Houston, USA). One tomographic scan was taken after cell assembly and a second scan after operating the cell for 30min at $100mA/cm^2$ while all other parameters were kept the same. The tubing and wiring were disconnected from the cell before taking the after-operation scan. Figure S10 shows exemplary tomographic slices that contain qualitatively the same degradation process as observed by the synchrotron XTM including cavities within the BPM and between BPM and anode CL as well as anode CL perforations. It is not possible to obtain time-resolved XTM due to the longer (>30min) acquisition for one scan. The image quality at the given labCT settings is furthermore lower compared to the synchrotron XTM with a larger voxel size, higher noise and lower contrast. Additionally, artifacts can be observed including black streaks and blurring of the catalyst layers ("catalyst shining") most likely due to the interaction of X-ray with heavier elements (Ir, Ag, Ti). It was consequentially not possible to employ the same quantitative image analysis as for the synchrotron XTM data. However, the qualitative analysis gives confidence in the general applicability of our results to CO_2 co-electrolysis.

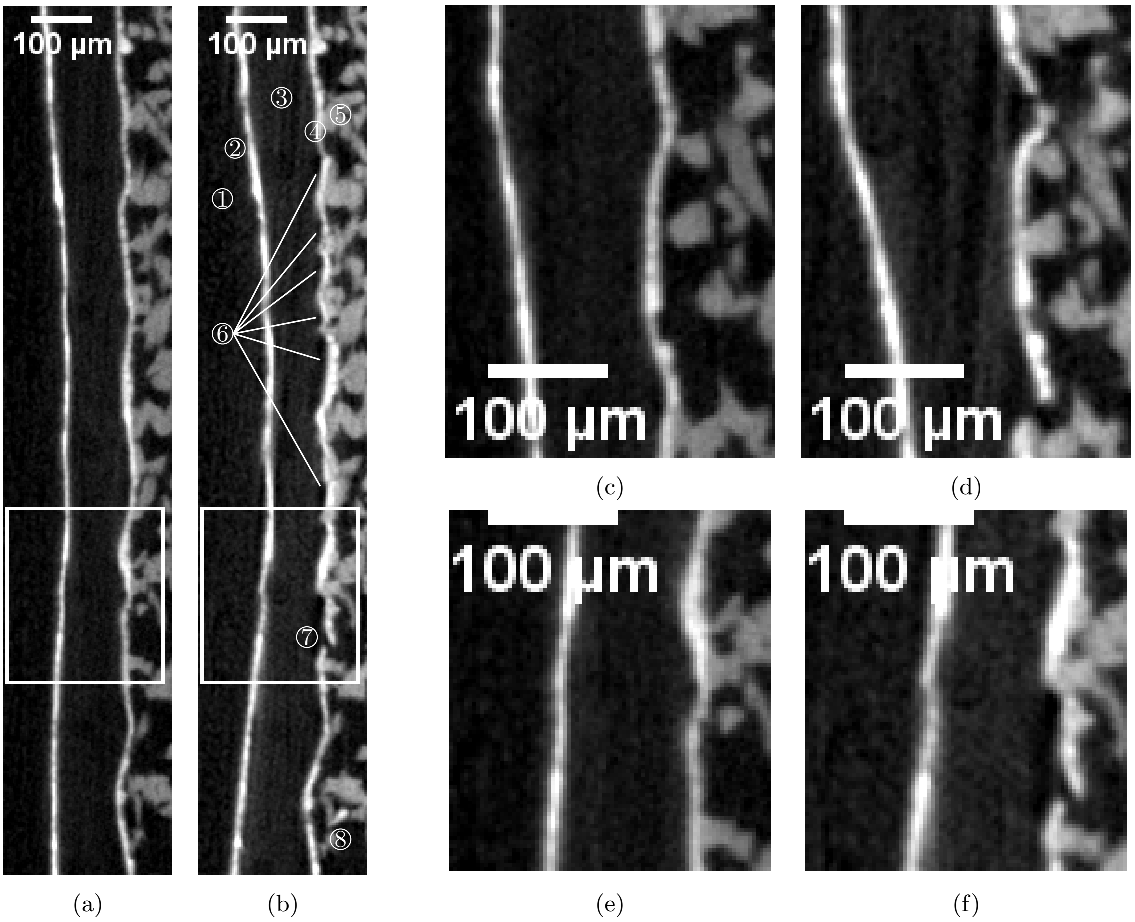

Figure S10: Tomographic slice of assembled cell with Ti fiber felt porous transport layer at anode. a) before operation, b) after operating at $100mA/cm^2$ for 30min with annotation: 1) carbon paper GDL, 2) cathode CL, 3) BPM, 4) anode CL, 5) Ti fiber, 6) cavities between BPM and anode CL, 7) anode CL hole, 8) mobilized piece of anode CL. c,d) Additional location at different slice showing anode CL perforation and membrane delamination. e,f) Close-up of white frame in a) and b)

S8 1D-model to understand CO_2 crossover

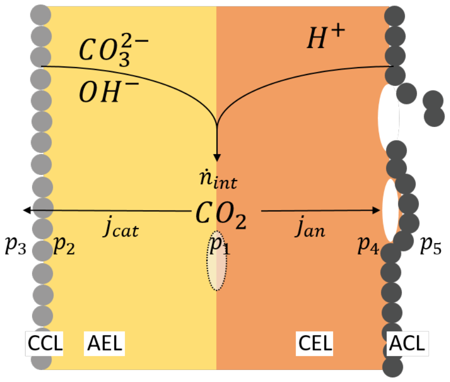

Figure S11: 1D schematic representation of ion recombination forming CO_2 at the junction of the bipolar membrane, resulting in CO_2 mass fluxes j_i and local CO_2 pressures p_i. n_{int} - rate of CO_2 production, CCL - cathode catalyst layer, AEL - anion exchange layer, CEL - cation exchange layer, ACL - anode catalyst layer

We devise a model for the CO_2 transport to discuss the obtained *operando* imaging results. The BPM in forward bias prevents direct CO_2 crossover to the anode in the form of (bi)carbonate ions by reaction at the BPM-junction with protons to CO_2 and water:

$$OH^- + H^+ \rightarrow H_2O \tag{S2a}$$

$$HCO_3^- + H^+ \rightarrow CO_2 + H_2O \tag{S2b}$$

$$CO_3^{2-} + 2H^+ \rightarrow CO_2 + H_2O \tag{S2c}$$

However, CO_2 and water can accumulate over time at the junction. We expect that the formation of gaseous CO_2 at sufficient concentration causes BPM delamination (figures 4-7) and damage to the anode CL (figures 4 and 8). An analytical model based on Fickian diffusion is used for first order approximation of CO_2 mass fluxes and concentrations at various locations in the MEA (figure S11) to complement the experimental observations of structural damages induced by gaseous CO_2.
CO_2 is produced at the BPM junction at a rate $\dot{n}_{int}\left[mol/cm^2 s\right]$

$$\dot{n}_{int} = \frac{I f_{CO_2}}{e N_A} \tag{S3}$$

where $I\left[mA/cm^2\right]$ is the current density, $e\,[C]$ is the elementary charge, $N_A\,[1/mol]$ is the Avogadro number and f_{CO_2} is the fraction of produced CO_2 per charge carrier. The formation of CO_2 depends on the different charge carriers in the AEM part of the bipolar membrane (reaction equation S2), i.e. HCO_3^- results in 1 molecule of CO_2 at the junction, while CO_3^{2-} in 0.5 CO_2 and OH^- in no CO_2 molecule per electron. The charge carrier distribution in the AEM part is complex and needs to be estimated based on other modeling work. We assume that all charge carriers are CO_3^{2-} in accordance to the current understanding of AEM[8, 2, 11, 7], i.e. $f_{CO_2} = 0.5$. For AEM, it is expected that with increasing current density, the CO_3^{2-} concentration is decreasing in favor of higher OH^- concentration[8, 11] and a recent study[10] shows an equivalent behavior of the BPM-AEL compared to a pure AEM[11]. The accumulating CO_2 at the junction leads to a concentration gradient $(c_i - c_j)$ and diffusive fluxes j_{ij} towards the cathode and anode, respectively, described by Fick's first law

$$j_{ij} = -\frac{D_{ij}^0 \epsilon_{ij} \left(c_i - c_j\right)}{\tau_{ij} \Delta_{ij}} = -D_{ij}^{eff} \left(c_i - c_j\right) \tag{S4}$$

where the subscripts i, j denote the layer interfaces 1-5 (figure S11), $D^0\left[m^2/s\right]$ is the diffusivity of CO_2 in the transport medium, ϵ the volume fraction of the transport medium, τ the tortuosity of the transport medium, $c\left[mol/m^3\right]$ the CO_2 concentration at the interface and $\Delta\,[m]$ the layer thickness. Accumulating CO_2 reaches the solubility limit and small pockets of gaseous CO_2 form at the interfaces, i.e. at the BPM junction and the membrane-CL interfaces. We consider virtual, zero-thickness, gas volumes at the interface with gas pressure $p_i\,[Pa]$. The CO_2 concentration c_i of dissolved CO_2 at the

interface is assumed to be in equilibrium with the gas phase. Dissolved CO_2 concentration and partial pressure are then related by the ideal gas law, which is approximately valid for CO_2 dissolved in water by a dimensionless Henry's number close to 1[9].

$$c_i \approx \frac{p_i}{RT} \tag{S5}$$

While CO_2 at the anode is instantly carried away by the water stream resulting in zero CO_2 pressure p_5, the partial CO_2 pressure at the cathode p_3 is known as

$$p_5 = 0 \tag{S6a}$$

$$p_3 = p_{amb} - p_{vap} \tag{S6b}$$

where p_{amb} is the ambient pressure and p_{vap} is the water vapor pressure depending on the set temperature T and relative humidity of the inlet CO_2 stream. Considering the conservation of mass and inserting (S5) in (S4) allows us to calculate the CO_2 crossover to the anode j_{an}, the back-diffusion to the cathode j_{cat} and the CO_2 pressure p_1 at the BPM junction at steady state.

$$0 = \dot{n}_{int} + j_{cat} + j_{an} \tag{S7a}$$

$$p_1 = \frac{RT\dot{n}_{int} + D_{cat}^{eff} p_3}{D_{cat}^{eff} + D_{an}^{eff}} \tag{S7b}$$

$$j_{cat} = -\frac{D_{cat}^{eff}(p_1 - p_3)}{RT} \tag{S7c}$$

$$j_{an} = -\frac{D_{an}^{eff}(p_1 - p_5)}{RT} \tag{S7d}$$

$$D_{cat}^{eff} = D_{13}^{eff} = \frac{D_{23}^{eff} D_{12}^{eff}}{D_{23}^{eff} + D_{12}^{eff}} \tag{S7e}$$

$$D_{an}^{eff} = D_{15}^{eff} = \frac{D_{45}^{eff} D_{14}^{eff}}{D_{45}^{eff} + D_{14}^{eff}} \tag{S7f}$$

The CO_2 flux is also influenced by the pressure drop across the cathode and anode CLs, which are calculated considering the permeability of the porous catalyst filled with gas (cathode) and water (anode) respectively. Since the crossover flux to the anode is equal to the flux across CEL and ACL, we can calculate the pressure drop at the anode CL

$$j_{an} = j_{14} = j_{45} \tag{S8a}$$

$$\Delta p_{ACL} = p_4 - p_5 = \frac{RT j_{an}}{D_{45}^{eff}} \tag{S8b}$$

and analogous for the cathode CL.

$$\Delta p_{CCL} = p_2 - p_3 = \frac{RT j_{cat}}{D_{23}^{eff}} \tag{S9}$$

The employed parameters are given in table S1. The model results for $I = 100 mA/cm^2$ are given in table S2.

Table S1: Parameters employed in the diffusive model. T=318K (45°C), 100% relative humidity of CO_2 gas stream. Thicknesses Δ are estimated based on the imaging results.

name	ij	D^0 $[m^2/s]$	ϵ	τ	Δ $[\mu m]$	comment
CCL	23	1.06 10^{-5}	0.5	1	8	self-diffusion of CO_2[12, 6] in porous, water free catalyst layer. ϵ and τ reasonable assumption based on[1].
AEL	12	3 10^{-9}	0.36	1.5	60	D^0 diffusivity of CO_2 in water[3, 5], ϵ water volume fraction of hydrated membrane estimated from inhouse gravimetric tests, τ assumption for well connected water domains[4, 13]
CEL	14	3 10^{-9}	0.36	1.5	60	ref. AEL
ACL	45	3 10^{-9}	0.39	2.8	10	D^0 diffusivity of CO_2 in water[5], ϵ and τ from nanotomography[1]

Table S2: Calculated CO_2 mass fluxes and pressures within the MEA for $I = 100mA/cm^2$.

variable	value	description
j_{cat}	0.336 $\mu mol/cm^2 s$	back diffusion to cathode
j_{an}	0.296 $\mu mol/cm^2 s$	crossover to anode
p_1	841 kPa	pressure at BPM junction
Δp_{23}	14 Pa	pressure drop across cathode CL
Δp_{45}	188 kPa	pressure drop across anode CL

References

[1] S. De Angelis, T. Schuler, M. Sabharwal, M. Holler, M. Guizar-Sicairos, E. Muller, and F. N. Buchi. Understanding the microstructure of a core-shell anode catalyst layer for polymer electrolyte water electrolysis. *Sci Rep*, 13(1):4280, 2023.

[2] S. Garg, C. A. Giron Rodriguez, T. E. Rufford, J. R. Varcoe, and B. Seger. How membrane characteristics influence the performance of co2 and co electrolysis. *Energy Environmental Science*, 15(11):4440–4469, 2022.

[3] K. H. Gertz and H. H. Loeschcke. Bestimmung des diffusionskoeffizienten von co2 in wasser. *Zeitschrift für Naturforschung B*, 11(2):61–64, 1956.

[4] A. Kusoglu and A. Z. Weber. New insights into perfluorinated sulfonic-acid ionomers. *Chem Rev*, 117(3):987–1104, 2017.

[5] W. Lu, H. Guo, I. M. Chou, R. C. Burruss, and L. Li. Determination of diffusion coefficients of carbon dioxide in water between 268 and 473 k in a high-pressure capillary optical cell with in situ raman spectroscopic measurements. *Geochimica et Cosmochimica Acta*, 115:183–204, 2013.

[6] T. R. Marrero and E. A. Mason. Gaseous diffusion coefficients. *Journal of Physical and Chemical Reference Data*, 1(1):3–118, 1972.

[7] A. Pătru, T. Binninger, B. Pribyl, and T. J. Schmidt. Design principles of bipolar electrochemical co-electrolysis cells for efficient reduction of carbon dioxide from gas phase at low temperature. *Journal of the Electrochemical Society*, 166(2):F34–F43, 2019.

[8] M. Ramdin, O. A. Moultos, L. J. P. van den Broeke, P. Gonugunta, P. Taheri, and T. J. H. Vlugt. Carbonation in low-temperature co2 electrolyzers: Causes, consequences, and solutions. *Industrial Engineering Chemistry Research*, 62(18):6843–6864, 2023.

[9] R. Sander. Compilation of henry's law constants (version 4.0) for water as solvent. *Atmospheric Chemistry and Physics*, 15(8):4399–4981, 2015.

[10] W. L. Toh, H. Q. Dinh, A. T. Chu, E. R. Sauvé, and Y. Surendranath. The role of ionic blockades in controlling the efficiency of energy recovery in forward bias bipolar membranes. *Nature Energy*, 8(12):1405–1416, 2023.

[11] L.-C. Weng, A. T. Bell, and A. Z. Weber. Towards membrane-electrode assembly systems for co2 reduction: a modeling study. *Energy Environmental Science*, 12(6):1950–1968, 2019.

[12] E. R. S. Winter. Diffusion properties of gases. part iv.—the self-diffusion coefficients of nitrogen, oxygen and carbon dioxide. *Trans. Faraday Soc.*, 47(0):342–347, 1951.

[13] Q. Zhao, P. Majsztrik, and J. Benziger. Diffusion and interfacial transport of water in nafion. *J Phys Chem B*, 115(12):2717–2727, 2011.
